# Inflammation Related to Obesity in the Etiopathogenesis of Gastroenteropancreatic Neuroendocrine Neoplasms

**DOI:** 10.3390/biomedicines10102660

**Published:** 2022-10-21

**Authors:** Marlena Budek, Jarosław Nuszkiewicz, Anna Piórkowska, Jolanta Czuczejko, Karolina Szewczyk-Golec

**Affiliations:** 1 Department of Medical Biology and Biochemistry, Faculty of Medicine, Ludwik Rydygier Collegium Medicum in Bydgoszcz, Nicolaus Copernicus University in Toruń, 24 Karłowicza St., 85-092 Bydgoszcz, Poland; 2 Department of Psychiatry, Faculty of Medicine, Ludwik Rydygier Collegium Medicum in Bydgoszcz, Nicolaus Copernicus University in Toruń, 9 M. Curie-Skłodowskiej St., 85-094 Bydgoszcz, Poland; 3 Department of Nuclear Medicine, Oncology Centre Prof. Franciszek Łukaszczyk Memorial Hospital, 2 Dr. I. Romanowskiej St., 85-796 Bydgoszcz, Poland

**Keywords:** adipokines, cytokines, gastroenteropancreatic neuroendocrine tumors, inflammation, neuroendocrine neoplasms, obesity

## Abstract

Gastroenteropancreatic neuroendocrine neoplasms (GEP-NENs) are rare neoplasms, which, due to their heterogeneous nature, non-specific symptoms, and lack of specific tumor markers pose many diagnostic and clinical challenges. In recent years, the effectiveness of GEP-NEN diagnosis has increased, which is probably associated with the greater availability of diagnostic tests and the cooperation of many experienced specialists in various scientific disciplines. In addition to the possible genetic etiology, the cause of GEP-NET development is not fully understood. Inflammation and obesity are known risks that contribute to the development of many diseases. Chronic inflammation accompanying obesity affects the hormonal balance and cell proliferation and causes the impairment of the immune system function, leading to neoplastic transformation. This review explores the role of inflammation and obesity in GEP-NETs. The exact mechanisms inducing tumor growth are unknown; however, the profile of inflammatory factors released in the GEP-NET tumor microenvironment is responsible for the progression or inhibition of tumor growth. Both the excess of adipose tissue and the impaired function of the immune system affect not only the initiation of cancer but also reduce the comfort and lifetime of patients.

## 1. Introduction

Infections caused by numerous pathogens (viruses, bacteria, parasites) or foreign bodies that cause chronic inflammation are classified as one of the risk factors for the development of cancer, such as stomach, liver, bladder, and gynecological cancer [1]. Infectious agents predisposing to the above-mentioned tumors include human papillomavirus, human immunodeficiency virus, hepatitis B or C virus, *Helicobacter pylori*, and *Schistosoma haematobium*, respectively [2]. Neoplasms located in the gastrointestinal tract and the pancreas are associated with chronic inflammation, but its causes are not fully understood [3]. However, it is known that pro-inflammatory and anti-inflammatory cytokines shape the tumor microenvironment and that tumor suppression or regression is dependent on cytokine levels [4]. Among obese patients, the disproportion between secretion of pro-inflammatory and anti-inflammatory factors disrupts the maintenance of homeostasis [5]. This contributes to an increase in the incidence of insulin resistance, type 2 diabetes mellitus, dyslipidemia, cardiovascular disease, non-alcoholic fatty liver disease, reproductive disorders, mental disorders, and cancer [5,6,7]. According to the International Agency for Research on Cancer, overweight and obesity may predispose to cancer of the esophagus, gastric cardia, colon and rectum, liver, gallbladder, pancreas, postmenopausal breast cancer, endometrial cancer, and ovarian, kidney, and thyroid cancer [8,9]. Metabolic abnormalities associated with chronic inflammation in adipose tissue may increase the risk of carcinogenesis by increasing tumor cell proliferation, inhibiting apoptosis, inducing angiogenesis, and damaging genetic material [10]. Changes in the profile of inflammatory factors in obesity and impaired function of the immune system could lead to the destruction of adipocytes and cell hypoxia, which is associated with chronic inflammation. This effect affects immune cells that infiltrate adipose tissue and secrete pro-inflammatory cytokines and chemokines [5,11]. Over the past decades, obesity has grown steadily and is becoming a global epidemic [12]. Obesity and the related chronic inflammation and metabolic dysfunctions are not only a factor predisposing to the development of cancer, but also a factor contributing to a worse well-being of patients and increasing mortality among people with cancer [13]. The following review aims to analyze the latest available literature on the effect of certain inflammatory factors in obesity and their apoptotic function in gastroenteropancreatic neuroendocrine neoplasms.

## 2. Gastroenteropancreatic Neuroendocrine Tumors

Gastroenteropancreatic neuroendocrine tumors (GEP-NETs) are a heterogeneous group of neoplasms originating from cells of the diffuse endocrine system (DES), located in the gastrointestinal tract and the pancreas [14]. Neuroendocrine cells exhibit mixed morphological and physiological characteristics of both nervous and endocrine systems [15,16]. Cells, scattered throughout the body, in response to neuronal or chemical stimulation, release hormones that control many of the body’s functions [17]. The incidence of neuroendocrine neoplasms (NENs) increased from 2.48 to 5.86/100,000/year in 1994–2009 and GEP-NETs account for 64–70% of them [18,19]. Over the past 40 years, the incidence of GEP-NETs has increased by about 6 times, making GET-NET the second most common cancer of the gastrointestinal tract, after colorectal cancer [20,21]. NEN of the gastrointestinal tract most often affects the small intestine, rectum, stomach, and appendix [22]. The reasons for the increase in successful diagnoses towards GEP-NETs are multifactorial and not entirely clear [23]. These include the influence of the external environment, dietary changes, but also increased access to screening tests, improved diagnostic techniques and advances in the histological evaluation of neuroendocrine tumors [24]. 

Patient response to treatment and survival is heterogeneous among patients with neuroendocrine neoplasms, and much evidence suggests a role of the systemic inflammatory response in the tumor microenvironment [25]. GEP-NETs are heterogeneous in terms of location, malignancy potential, prognosis, treatment methods, and functionality, which also makes their diagnosis difficult [26,27]. Diagnosis of GEP-NETs often takes place only at the time of an advanced disease or in the process of metastasis (5–7 years on average), which significantly delays effective therapy and increases the probability of metastases [27,28]. About 50% of GEP-NETs, previously called carcinoids, are identified unexpectedly during surgery [18]. Usually the detection of distant metastases, mainly in the liver or lymph nodes, is the basic diagnostic factor that reduces the survival of patients with ongoing metastatic process as compared to patients with primary GEP-NET changes without metastasis [27]. In addition to incidental tumors, there are fully symptomatic tumors associated with the primary location of the tumor or the secretion of serotonin and other hormones by carcinoids in the process of metastasis [29]. The clinical manifestation of the disease depends on whether the tumor exhibits endocrine activity or is endocrine inactive [30]. Endocrine active tumors that secrete biogenic amines or polypeptide hormones can cause a variety of clinical syndromes, including Zollinger–Ellison, Verner–Morrison, insulinoma, glucagonoma, and carcinoid syndrome caused by endogenous serotonin release [30,31]. The symptoms of carcinoid syndrome include facial flushing, diarrhea, vomiting, bronchospasm, wheezing, and may be associated with increased morbidity and mortality [29,31,32]. The cause of neuroendocrine tumors is not fully understood, but the risk is significantly increased in people who inherit certain genetic syndromes, including multiple endocrine neoplasms type 1 (MEN1), neurofibromatosis, von Hippel–Lindau syndrome, and tuberous sclerosis [33]. Primary changes in GEP-NETs are most often located in the gastric mucosa, small intestine, large intestine, rectum, and pancreas [18].

## 3. Biochemical Diagnosis of GEP-NET

GEP-NET diagnostics is complex and often ambiguous due to the heterogeneous clinical manifestation of neoplasms. Diagnostic methods used include medical and physical examination, biochemical analysis, pathomorphological analysis, and radioisotope diagnostics [28]. GEP-NET cells have the ability to synthesize, store, and secrete biogenic peptides, hormones, and amines, which are used in biochemical diagnostics of GEP-NETs, in monitoring radical surgery or pharmacological treatment and in prognosis [34]. Among the specific markers effective in the diagnosis of certain types of GEP-NETs, which simultaneously confirm the patient’s clinical state, insulin (in insulinoma), gastrin (in gastrinoma), glucagon (in glucagonoma), vasoactive intestinal peptide (in VIPoma), and somatostatin (in somatostatinoma) should be mentioned [35]. In the case of carcinoids, excretion of 5-hydroxyindole acetic acid (5-HIAA) in the 24-h urine collection is useful in diagnosis and treatment monitoring [36]. General markers used to screen patients without characteristic symptoms of hormone overproduction include chromogranin A (CgA), neuron-specific enolase (NSE), pancreatic polypeptide, and glycoprotein hormone subunits [34,37]. Among the above-mentioned parameters, the most frequently used marker in the diagnosis of GEP-NET is CgA, which is expressed in 80–90% of patients with neuroendocrine tumors [34,37,38]. CgA is an acid glycoprotein found in a variety of neuroendocrine tissues including the adrenal medulla, gastrointestinal tract, pancreas, parathyroid glands, reproductive system, adipocytes, cardiovascular, and immune systems [39]. 

Many publications indicate that the dysregulated release of CgA by neuroendocrine cells may affect the components of the tumor stroma, contributing to the regulation of tumor growth or progression [40]. Both the mass and the secretory activity of the tumor should be taken into consideration when interpreting the CgA level measured [41]. Even several hundred times higher CgA values were recorded in carcinoid tumors and in the cases of liver metastases [42]. CgA in midgut NETs may serve as an independent prognostic factor for patient survival. The sensitivity of the parameter varies depending on the tumor location between 10–100%, with the highest sensitivity observed in gastrinoma, glucagonoma, and carcinoid tumors, whereas the CgA specificity is 68–100% [42]. The analysis by Yang et al. [43] showed that CgA is an effective biomarker for the diagnosis of NET with a sensitivity of 73% and a specificity of 95% and may be helpful in the control of NET treatment. Comparative studies by Lyubimova et al. [44] on a group of patients with NETs of various localization and healthy people showed diagnostic sensitivity and specificity at the level of 85.8% and 98.5%, respectively. The highest concentrations of CgA have been found in patients with gastric and lung NETs, while the highest median values of CgA have been found in patients with tumors of the small intestine, colon, and pancreas (about 12, 11, and 8 times higher than in the controls, respectively), especially in the case of patients with liver metastases and carcinoid syndrome [44]. 

In the case of patients with GEP-NET, Chou et al. [45] determined the sensitivity of CgA at the level of 86% and the specificity at the level of 88%. In some patients with stable disease, an approx. 20% decrease in CgA levels after treatment compared with the initial values has been observed [45]. Literature data show that CgA is a promising marker in the diagnosis of GEP-NET; however, the combination of chromogranin with other parameters seems to be much more promising. Simultaneous determination of CgA and NSE has been shown a higher sensitivity than the independent determination of each of these parameters [41]. In the diagnosis of pancreatic NETs, the determination of CgA and pancreatic polypeptide is significantly useful [46]. The use of CgA has many advantages, such as simplicity and availability of the method and minimally invasive collection of biological material (blood serum sample). These properties make it possible to monitor the CgA level in patients frequently [43]. The meta-analysis by Al-Risi et al. [39] showed that CgA is an effective and non-invasive marker for the clinical detection and control of NET treatment, taking into account other patient’s complaints or medications, mainly proton pump inhibitors. NSE, which is a highly specific marker of neurons and peripheral neuroendocrine cells, is currently the most reliable marker in the diagnosis and prognosis of small cell lung cancer, although it may also be useful in the diagnosis of GEP-NET [47]. Studies by Nobels et al. [48] showed that NSE is elevated in 31–44% of GEP-NET patients. The above information demonstrates the need to find new specific markers for risk assessment, diagnosis, and treatment control of different types of NENs.

## 4. The Endocrine Role of Adipose Tissue and Inflammation in Obesity

In recent decades, numerous information has been discovered about adipose tissue relating to its biology and biochemistry [49]. It has been found that it is not only an energy store, but also an endocrine gland that releases active compounds involved in metabolic changes and the body’s immune responses [50]. Adipose tissue, classified as a connective tissue, consists of adipocytes, which are the main depot of triacylglycerols, preadipocytes, and other components, such as connective tissue stroma, vascular stromal cells, cells of the immune system, or nerve cells [51,52]. Adipose tissue in a human body consists mainly of white adipose tissue (WAT) and brown adipose tissue (BAT), which differ in their morphology and function of adipocytes [53]. BAT, which occurs mainly in the neonatal period, specializes in maintaining physiological body temperature [54]. It is a highly oxidative tissue containing adipocytes with numerous mitochondria that generate energy in the form of heat by oxidation of fatty acids via uncoupling protein 1 (UCP1) [55]. WAT is located subcutaneously or peritoneally and is the main energy storage [56]. WAT is responsible for the storage of triacyl glycerides during increased energy supply and for their use during Increased energy demand such as malnutrition [57]. 

The World Health Organization (WHO) defines obesity as an excessive and abnormal accumulation of adipose tissue, which is associated with a negative impact on human health [58]. Chronic inflammation in adipose tissue in obesity, referred to as low-grade inflammation, is mainly caused by a disturbed balance in the secretion of pro- and anti-inflammatory cytokines and adipokines [59]. Excessive supply of macronutrients enhances the release of numerous inflammatory mediators by adipose tissue [60]. Increased release of pro-inflammatory factors positively correlates obesity with chronic inflammation and may result in insulin resistance, metabolic syndrome, dyslipidemia, hyperglycemia, and hypertension [61,62]. Pro-inflammatory substances include tumor necrosis factor α (TNF-α), interleukins 1β (IL-1β) and 6 (IL-6), C-reactive protein (CRP), plasminogen activator inhibitor (PAI-1), monocyte chemotactic protein 1 (MCP-1), intercellular adhesion molecule 1 (ICAM), leptin, visfatin, resistin, or sex hormones [63,64]. These pro-inflammatory cytokines regulate adipocyte proliferation and apoptosis, induce lipolysis, inhibit lipid synthesis, and lower blood lipids through autocrine and paracrine mechanisms [64]. On the other hand, the excess of adipose tissue contributes to the reduction of the production of anti-inflammatory substances, which include mainly adiponectin, soluble frizzled-related protein 5 (SFRP5), which inhibits the Wnt signaling pathway, an antagonist of the IL-1 receptor, as well as interleukins IL-4, IL-10, and IL-13 [60,61,63,65]. The overproduction of adipocytokines by the quantitatively and volumetrically increased cells of adipose tissue causes inflammation to become chronic. The process also includes enhanced oxidative stress, which leads to a number of mechanisms involved in carcinogenesis [60,66]. 

Leptin, which had been considered to be a substance that could support the treatment of obesity by suppressing appetite, reducing adipose tissue gain and inducing metabolism and thermogenesis, has become the best-known pro-inflammatory adipokine, having the features of a strong mediator of acute inflammation [67]. It is associated with the formation of a leptin-resistance mechanism, which takes place, among others, due to an impaired transport of leptin through the blood–brain barrier, inhibition of leptin signaling pathways in the hypothalamic centers and the polymorphism in Ob gene, responsible for leptin synthesis [68]. Inflammation of adipose tissue caused by an increased supply of nutrients is associated with an increased apoptotic potential of adipocytes [69]. Apart from adipocytes, other structures that build adipose tissue also have immunomodulatory properties. These include preadipocytes, fibroblasts, lymphocytes, mast cells, blood vessel wall cells, eosinophils, and macrophages [70,71]. Infiltrating macrophages, which release into the circulation further pro-inflammatory mediators (e.g., IL-6, IL-8, TNF-α) along with harmful products of intense oxidative stress, which are also released from damaged blood vessels, increase adipocyte hypoxia and lipolysis, promoting the persistence of inflammation [62,72]. The negative influence of the interaction between macrophages and cells of the immune system on the cells of peripheral tissues influences the development of many metabolic and immune diseases, including the development and progression of cancer [73].

## 5. Inflammation in Carcinogenesis

More and more data indicate the participation of inflammation in carcinogenesis, as evidenced by the formation of neoplasms at the site of infection, irritation, or chronic inflammation [74]. In the tumor microenvironment, apart from neoplastic cells, the presence of immune cells, fibroblasts, endothelial cells, growth factors, pro-angiogenic factors, as well as cytokines and chemokines has been demonstrated [75]. Components within the tumor may predispose to the neutralization of malignant tumor cells, but as a result of oncogenic evolution, transformed cells may escape from the control of the immune system and then antitumor activity is suppressed [75,76]. If the elimination of tumor cells by the immune system is no longer possible, cells and factors forming the tumor microenvironment can help tumor cells to survive and avoid apoptosis, induce angiogenesis, as well as stimulate metastasis [75,77]. Intensive production of reactive oxygen and nitrogen species, which is enhanced by immune cells infiltrating the inflammatory focus, may damage normal tissues and, consequently, initiate compensatory cell proliferation [78]. These processes can lead to the duplication and accumulation of DNA damage, gene mutations and the stimulation of internal and external carcinogens [78]. DNA damage can also be caused by cytokines, for example IL-22, which activates the response to abnormal DNA by regulating the expression of numerous genes [79]. 

Every inflammatory factor has specific functions in the stages of neoplastic tumor development, namely at the stage of initiation, promotion, progression and metastasis [80]. The most important association between cancer and inflammation is related to the activity of the nuclear factor kappa-light-chain-enhancer of activated B cells (NF-кB), the signal transducer 3 (STAT3) pathway and an activator of transcription [81]. These signaling pathways control genes necessary for angiogenesis and affect the ability of cancer cells to metastasize [82,83]. Cytokines released by inflammatory, immune, and neoplastic cells may have pro-cancer, but also anti-tumor effects, depending on their profile [4]. TNF-α, IL-6, and IL-17 primarily accelerate tumor progression, while cytokines normally leading to the tumor suppression include, among others, pro-apoptotic ligand inducing apoptosis associated with TNF (TRAIL, TNF-related apoptosis-inducing ligand), anti-inflammatory IL-10 and IL-12, activating cytotoxic T lymphocytes and NK cells, and regulating the expression of cytotoxic mediators [4,81]. Transforming growth factor beta (TGF-β) and IL-23 may play a role in both promoting tumor progression and suppressing tumor [4].

### 5.1. Interleukin 6

IL-6 is secreted mainly by monocytes, and also by macrophages, Kupffer cells, keratinocytes, endothelial cells, B and T cells, and cancer cells [84]. Increased production of IL-6 is the result of infection or tissue damage, and it performs its functions by binding to the IL-6 receptor [84,85]. This cytokine has pleiotropic effects including acute phase protein production, hematopoiesis, osteoclast activation, proliferation, and differentiation of B lymphocytes to produce antibodies [86]. Pro-tumor activity has been shown in mouse plasmacytoma and human myeloma, in cancer of the lung, breast, colon, prostate, ovary, pancreas, lung, cervix, and renal cell carcinoma [87]. IL-6 enhances its action promoting the survival of neoplastic cells by activating IL-6/STAT3 signaling [88]. It is of major importance in promoting tumor growth, it induces the release of reactive oxygen and nitrogen species, and is a suppressor of apoptosis [79,84,89]. IL-6 enhances the proliferation of colorectal cancer cell lines in vitro, resulting in an increase in NF-kB and STAT3, which induce colorectal cancer cell growth. Inhibition of STAT3 in intestinal epithelial cells effectively stopped tumor induction and growth [84,90].

### 5.2. Tumor Necrosis Factor Alpha

TNF-α, released mainly by monocytes and lymphocytes is a multi-directional cytokine [91]. As a pro-inflammatory cytokine, it may be involved in inflammatory-related cancer induction, survival, proliferation, differentiation, and even in cell death [92]. By inducing genes encoding nuclear factor kappa-light-chain-enhancer of activated B cells (NF-κB) dependent anti-apoptotic molecules, it promotes the survival of cancer cells. By permanently activating the N-terminal c-JUN (JNK)-dependent signaling pathway through stimulation of the JNK-phosphorylating kinase complex, mitogen-activated protein kinases (MAPKs) contribute to cell death [93]. In addition, the pro-apoptotic function of TNF-α is enhanced by the production of reactive oxygen and nitrogen species [94]. It has been suggested that the properties of TNF contributing to the death of neoplastic cells may constitute a potential cancer therapy [92].

### 5.3. Interleukin 17

IL-17 is a potent pro-inflammatory cytokine released by T lymphocytes, type 3 innate lymphoid cells, δγT lymphocytes, and NK cells [95]. IL-17 may participate in both the early and late stages of cancer development and in its growth, as well as it may contribute to the initiation of metastatic processes [96]. It is an important pro-inflammatory cytokine enhancing the release of numerous pro-inflammatory cytokines and chemokines (TNF-α, IL-6, IL-1β) and thus intensifying inflammation [4]. IL-17 activates the Src/PI3K/AKT/NF-κB, MAPK, STAT3, and Cox-2 pathways that play a significant role in oncogenesis, angiogenesis, and metastasis. Additionally, it induces tumor growth and angiogenesis [95,96].

### 5.4. Tumor Necrosis Factor-Related Apoptosis-Inducing Ligand

TRAIL can be expressed by activated T lymphocytes, NK cells, dendritic cells, and macrophages [97]. It is a molecule that belongs to the TNF superfamily and binds to cell surface receptors, namely DR4 (TRAILR-1) and DR5 (TRAIL-2) [98]. Forming a death-inducing signaling complex (DISC), it triggers the apoptotic cascade independently from p53 [99]. It is a powerful anti-cancer agent, inducing apoptosis in various types of cancer cells, while having low impact on healthy cells [100]. However, it is not a perfect anticancer drug because malignant neoplasms develop mechanisms of resistance to apoptosis, which significantly hinders effective therapy [99,101].

### 5.5. Interleukin 10

IL-10 is an immunosuppressive cytokine with a strong anti-inflammatory effect, produced mainly by regulatory T cells, helper T cells, as well as monocytes, mast cells, activated T, and B lymphocytes [102]. Lack of IL-10 is associated with the occurrence of autoimmune and inflammatory diseases, as confirmed by experimental studies in the model of IL-10 deficient mice, in which spontaneously developed enteritis and administration of recombinant IL-10 showed therapeutic efficacy [103]. IL-10 inhibits tumor growth and progression, modulates apoptosis, and inhibits angiogenesis during tumor regression [104]. However, it is possible that this cytokine plays a dual role in oncogenesis. At the time of tumor formation, it stimulates the death of cancer cells, but for cancer cells that have escaped from the control of the host’s immune system, IL-10 may be a strong cancer promoter [105]. Activation of the STAT3 pathway and induction of anti-apoptotic genes play a role in the proliferation and survival of neoplastic cells [4].

### 5.6. Interleukin 12

IL-12 is a potent, pro-inflammatory cytokine released by antigen presenting cells, usually in response to microbial pathogens [106]. IL-12 receptors are mainly expressed on CD8 + T cells, NK cells, and NKT cells and they significantly affect the activity of this cytokine [107]. L-12 is responsible for a wide variety of functions, such as induction and enhancement of cellular immunity, induction, and differentiation of TH1 cells, enhancement of T and NK cell activation and cytotoxic capacity, inhibition of tumor-associated immunosuppressive cells, and myeloid suppressor cells [108]. IL-12 shows its anti-tumor activity also by increasing the production of interferon γ (IFN-γ), which is an anti-angiogenic, cytostatic, and cytotoxic molecule; it can also induce apoptosis of cancer cells and control tumor growth [106,108].

### 5.7. Transforming Growth Factor β

Transforming growth factor β (TGF-β) is produced mainly by macrophages and neutrophils. Like IL-6 and TNF-α, it is involved in cancer stromal activation, immune escape, angiogenesis, migration, and differentiation of many cell types and inhibition of apoptotic pathways [109,110]. TGF-β acts as a potent tumor suppressor in normal and precancerous cells by inhibiting the G1 phase of the cell cycle, inducing apoptosis in early cancer and autophagy in some cancer cells. In the later stages of cancer, when tumor cells have acquired oncogenic mutations and/or have lost gene suppressor function and have become resistant to TGF-β-induced growth arrest, this cytokine leads to tumor promotion by inducing epithelial–mesenchymal transition (EMT) [111,112]. The primary signaling pathway of TGF-β is the binding of the cytokine to the TGF-βR receptor (TGF-βRI or TGF-βRII) and subsequent activation of the transcription factors Smad. There are other mechanisms of action of TGF-β, e.g., it induces rapid activation of extracellular kinase regulated by Ras signals (Erk), acts through TGF-β-activated kinases (MAPK), namely 4-c-Jun kinase N-terminal (TAK1-MKK4-JNK), TAK1-MKK3/6-p38, Rho-Rac-cdc42, and the signaling pathway of phosphatidylinositol 3-kinase (PI3K) and Akt protein kinase [113].

**Figure 1:**
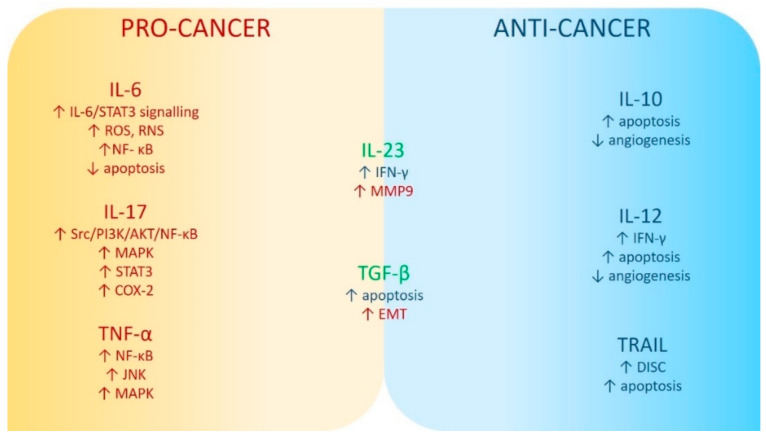
Mechanisms of pro- and anti-carcinogenic activity of selected inflammatory factors. Abbreviations used: Akt (PKB): protein kinase B; COX2: cyclooxygenase 2; DISC: death-inducing signaling complex; EMT: epithelial–mesenchymal transition; IL-6: interleukin 6; IL-17: interleukin 17; IL-23: interleukin 23; IFN-γ: interferon-gamma; JNK: c-Jun N-terminal kinases; MAPK: mitogen-activated protein kinases; MMP9: matrix metallopeptidase 9; NF-кB: nuclear factor kappa B; PI3K: phosphatidilinositol 3-kinases; RNS: reactive nitrogen species; ROS: reactive oxygen species; Src (short from sarcoma): proto-oncogene tyrosine–protein kinase; STAT3: signal transducer and activator of transcription 3; TNF-α: tumor necrosis factor α. The upwards arrow indicates an increase in the parameter’s level; the downwards arrow indicates a decrease in the parameter’s level.

Figure 1 summarizes possible pro- and anti-carcinogenic mechanisms of selected inflammatory factors.

## 6. Inflammatory Factors in GEP-NET

GEP-NETs are often associated with the occurrence of chronic inflammation, which probably stimulates neuroendocrine cells in the gastrointestinal mucosa to hyperplasia and neoplastic transformation [114,115]. TNF-α, IL-2, IL-6, IL-1β, and TGF-β play a special role in creating favorable conditions for the development of GEP-NETs [116,117]. Berkovic et al. [114] showed in their studies a relationship between TNF-α and IL-2 and the induction of GEP-NET. TNF-α and IL-2 influence was significantly associated with single nucleotide polymorphisms for these cytokines. It was also determined that those cytokines could be more sensitive markers for both functional and non-functional GEP-NETs (NF-GEP-NETs) than the routinely used CgA [114,116]. Berkovic et al. [117] conducted additional studies on the possible contribution of other cytokines to the predisposition to neuroendocrine neoplasms of the gastrointestinal tract and pancreas. The study showed a correlation between the occurrence of the IL-6-174 C/G genotype and the development of GEP-NET. No differences were found between the distribution of other investigated genotypes in patients with GEP-NET compared to the control, healthy group. However, it was noted that high expression of genotypes (CG and GG) was more common in patients with non-functional pancreatic neuroendocrine tumor (pNET), which may be a factor differentiating functional pNET from non-functional pNET. Elevated levels of IL-6 have been significantly correlated with the GG IL-6-174 polymorphism in all GEP-NETs [117]. IL-6, which is a pleiotropic cytokine likely to be released in many types of cancer (e.g., colorectal cancer, advanced gastric cancer, and others), may act as an auto- or paracrine growth factor and may promote tumor invasiveness and metastasis [117,118,119,120]. It has been proven that polymorphisms in genes for both IL-6 and IL-1β are involved in the development of neuroendocrine tumors [114]. The extent to which the above-mentioned cytokines are involved in the neoplastic transformation of neuroendocrine cells depends on the type of genotype. The genotypes IL-1β −511/+3954 CTCC and IL-6-174 CG/GG are associated with an increased susceptibility to the development of non-functioning NENs [116,121,122]. It has been also shown that carriers of the IL-1β −511/+3954 CTCT genotype are exposed to functional pNET [121]. Mahečić et al. [116] determined high IL-6 expression and low IL-1β and IL-2 expression in most GEP-NETs. They also found that TNF-α is elevated in GEP-NETs, especially in tumors with increased proliferation (more advanced). TNF-α concentrations positively correlated with the death rate [116].

TGF-β is a protein actively secreted by tumor cells that controls proliferation, contributing to invasion and metastasis while reducing the host’s immune response to the tumor [123]. Its effect on cell proliferation and metastasis processes is complex [124]. In neoplasms originating from epithelial and nerve cells, in the first phase, TGF-β serves as a tumor growth suppressor. In the later stages of the disease, the signaling between TGF-β and somatostatin is disrupted (somatostatin pathway is an antiproliferative pathway) forming an increased metastatic potential, in which TGF-β becomes an inducer rather than a suppressor of the transformation of neuroendocrine tumors [124,125]. Wimmel et al. [126] detected the expression of TGF-β1 and its receptors (TGF-βRI and TGF-βRII) in three tested NET cell lines and in 67% of human NETs. Three lines of neuroendocrine cells, namely BON, LCC-18, and QGP, were used for the study, whereas two lines of exocrine pancreatic cancer cells (BxPc3 and PANC-1), sensitive to TGF-β1, were used as a positive control. In the case of BON and LCC-18 cell lines, the treatment with TGFβ-1 caused the transactivation of the TGFβ-responsive reporter construct, as well as c-myc inhibition and induction of p21(WAF1), a cyclin-dependent kinase inhibitor. TGFβ-1 inhibited anchorage-dependent and time- and dose-independent increase in TGFβ-1 responsive cell lines resulting in arrest of the G1 phase of the cell cycle, with no evidence of apoptosis. Functional inactivation of endogenous TGF-β revealed the existence of an autocrine antiproliferative loop in neuroendocrine cells [126]. TGF-β1 and receptors for this cytokine are expressed in most GEP-NETs. The study by Wimmel A. et al. [126] have proven that NET cells may be sensitive to autocrine and paracrine antimitogenic effects of TGF-β1.

The pathogenesis of gastric neuroendocrine tumors (gNET) is associated with chronic autoimmune atrophic gastritis. In gNET, the production of antibodies against the parietal cells of the stomach or an intrinsic factor leading to Addison–Biermer anemia has been observed [127,128]. The role of IL-2 in the regulation of the neuroendocrine system and the release of gastrointestinal hormones has been also confirmed [129]. Studies by Pavel et al. [130] have shown that vascular endothelial growth factor (VEGF) and IL-8 are associated with tumor progression and may be qualified as markers of prognosis and treatment control in patients with neuroendocrine neoplasms. These results point that anti-angiogenic therapies should be also considered in the treatment of patients with GEP-NET. Although normal pancreatic cells do not express IL-8, pNENs show increased expression of IL-8 and its receptors, which in turn suggests the possibility of regulating tumor behavior through auto- or paracrine loops of the IL-8 signaling pathway [131]. In the study by Berkovic et al. [132], a strong expression of VEGF-A in pNENs and low-grade GI-NENs has been observed, which has been associated with a better prognosis of the patient. Berardi et al. [133] proved that single nucleotide polymorphisms in VEGF-A and its VEGFR-3 receptor correlate with poor prognosis in GEP-NEN, which may make it a potential prognostic factor in the clinical management of GEP-NEN. VEGFR-3 is the main regulator of proliferation, migration, and apoptosis of lymphatic cells and its expression is increased during angiogenesis. Therefore, changes in VEGF and VEGFR-3 found in GEP-NET may induce the angiogenesis process and affect the malignancy of the tumor [133].

In recent years, indicators, such as neutrophil–lymphocyte ratio (NLR) and platelet–lymphocyte ratio (PLR), calculated on the basis of inflammatory markers circulating in the blood, have aroused interest [134]. An association between high NLR and tumor size, stage, high grade, shorter survival in NEN patients has been observed. Thus, NLR may be a potential independent marker of nodal metastasis and relapse-free survival [135,136]. Pozza et al. [137] in their studies on a group of patients with NET of the anterior, middle, and posterior intestine have shown a significant importance of NLR value in patients with distant metastases, mainly to the peritoneum, while PLR and platelet-to-white-blood ratio (PWR) were not associated with the metastasis process but showed a predisposition to detect multifocal disease with varying sensitivity and specificity. However, some studies do not support the importance of PLR as a predictor of the presence of metastases or multifocal disease [137]. Kulahci et al. [134], based on retrospective studies performed in 2014–2020 on patients with NETs, found a correlation between a higher NLR value (cut-off value above 3.01) and tumor location, higher histological grade, increased mitosis, higher Ki-67 proliferation index, metastases, and lymphatic involvement. In the case of PLR, no such correlations have been observed. Until 2021, there have been many retrospective studies indicating the prognostic value of NLR and PLR indicators. However, in order to be able to use these indicators as strong predictors of NETs, they should be confirmed in prospective studies on larger study groups [138]. 

Table 1 presents the results of selected studies investigating the role of inflammation in GEP-NETs.

## 7. Obesity and Its Comorbidities and the Risk of GEP-NETs

**Figure 2:**
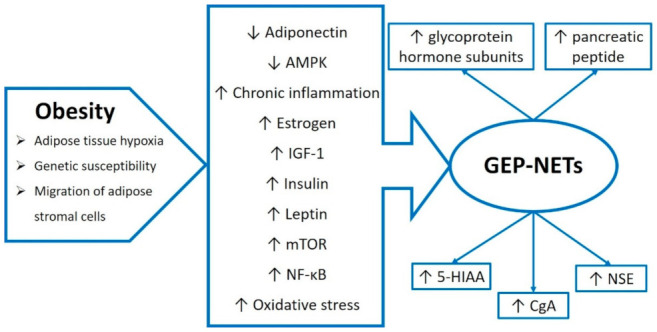
Relationship between obesity and gastroenteropancreatic neuroendocrine tumors (GEP-NETs). Abbreviations used: 5-HIAA: 5-hydroxyindole acetic acid; AMPK: AMP-activated protein kinase; CgA: chromogranin A; IGF-1: insulin-like growth factor-1; mTOR: mammalian target of rapamycin kinase; NF-κB: nuclear factor kappa B; NSE: neuron-specific enolase. The upwards arrow indicates an increase in the parameter’s level; the downwards arrow indicates a decrease in the parameter’s level.

Obesity and the metabolic syndrome are associated with a significant negative impact on the health and condition of an organism, being a risk factor for chronic diseases and cancer [141]. In addition to the inflammation, carcinogenic mechanisms in obesity include excessive estrogen, hyperinsulinemia, increased insulin-like growth factor-1 (IGF-1) secretion, impaired release of adipose tissue hormones with proliferative (e.g., leptin) or anti-proliferative (e.g., adiponectin) effects, direct or indirect influence of adipocytes on cell growth regulators, including mammalian target of rapamycin kinase (mTOR) and AMP-activated protein kinase (AMPK), as well as an altered immune response affecting NF-κB and inducing oxidative stress [142,143,144,145]. The possible links between obesity and GEP-NETs are presented in Figure 2.

Keshishian et al. [146] in 2002 noticed an increased incidence of carcinoids in obese people (1.5%) compared to the general population, which increased interest in the influence of obesity on neuroendocrine tumors. New hypotheses for obesity and cancers have emerged, such as excessive adipose tissue hypoxia, genetic susceptibility, and migrating adipose stromal cells [145]. In the years 1967–2010, a group of adolescents (2.3 million) aged 16–19 was examined in terms of the impact of obesity and sociodemographic factors on the risk of GEP-NET [147]. The analysis of the results showed a statistically significant association between increased body mass, increased body mass index (BMI), and the risk of gastric NETs [147]. In another case-control study, Hassan et al. [148] showed that, in females, type 2 diabetes mellitus, developed at least one year before cancer diagnosis (information based on history), has been a statistically significant factor of the risk of gastric NETs. However, long-term diabetes did not affect the development of pancreatic NETs. All patients in the study group with gastric NETs had diabetes >1 year before the diagnosis of NETs, which is an 8-fold higher risk of this type of NET compared to healthy women in the control group [148]. Although the mechanisms by which diabetes could induce gastric NETs are not clear, it is possible that it stimulates the chronic inflammation of the gastric mucosa, its disappearance, and thus increased oxidative stress, causing mutations in DNA [149]. The risk of pancreatic NETs in both male and female was observed only in people with type 2 diabetes mellitus diagnosed within 1 year of NET detection [148]. Obesity predisposes to insulin resistance and type 2 diabetes mellitus; therefore, the influence of excess body mass on the development of GEP-NET tumors cannot be excluded [150]. Hassan et al. [148] determined the BMI of patients at the time of NET diagnosis without taking into account the fluctuations in body mass before diagnosis. Due to the often-late diagnosis of GEP-NETs, some subjects may have experienced weight loss due to the progressive disease; therefore, the association of diabetes in obesity with the development of GEP-NETs cannot be excluded [148]. The aim of the study by Santos et al. [151] was to determine the association between obesity, the metabolic syndrome (MetS), and the development of GEP-NET. The authors have indicated that patients who met more of the MetS diagnostic criteria were more likely to develop GEP-NETs [151]. The factors of MetS, especially visceral obesity, dyslipidemia, increased waist circumference, and increased fasting glucose, correlated with the occurrence of GEP-NETs [151]. In a cohort study conducted by Santos et al. [152], hormonally active gastrointestinal neuroendocrine tumors (GI-NETs) with carcinoid syndrome have been associated with MetS in 48.1%. More than 50% of patients with progressive metastasis had features of MetS. Few analyses have been carried out to assess the impact of increased BMI on the risk of pancreatic neuroendocrine tumors and they are inconclusive, as evidenced by the meta-analysis published in 2015 [153]. Studies by Zhan et al. [154] and Halfdanarson et al. [155] showed an increased BMI in pNEN patients compared to the control group, but the differences were minor. These results do not allow for an unequivocal explanation of the relationships between obesity and the risk of pNEN. Interestingly, case-control studies by Hassan et al. [148] showed an inverse dependency between overweight or obesity and NETs of the small intestine, while the cohort studies by Cros et al. [156] demonstrated an increased risk of NETs of the small intestine in the group with the highest BMI compared to patients with the lowest amount of adipose tissue. 

The analysis of data from the Nationwide Inpatient Sample (NIS) by Glazer et al. [157], which concerned the association between obesity and the incidence and mortality of patients with abdominal NETs, showed that obesity is strongly associated with a reduced death rate (*p* = 0.02) and that malnutrition may be associated with a 5-fold higher risk of hospital mortality (*p* < 0.0005). The above analysis was carried out on the basis of information about patients from the NIS database from January 1, 2009 to December 31, 2010. Interestingly, this analysis shows that obesity may have a protective effect against NET severity [157]. Similar conclusions were drawn on the basis of the assessment of the impact of baseline BMI (at the stage of disease diagnosis) on the treatment outcomes of patients with NEN and it has been proven that higher BMI is associated with better overall survival among patients with NEN [158]. These analyzes were carried out on the basis of the combined administrative databases of one of the provinces of Canada from 2004–2019, reviewing patients with NEN and data on the BMI of patients at the time of diagnosis of NET [158]. Interestingly, Pareira et al. [159] found the presence of IL-6 in the peritumoral stroma of the intestine and pancreas (mainly in fibroblasts, endothelium, and immune cells), but there were not statistically significant differences between GI-NET patients with MetS and without MetS. However, it has been shown that peritumoral IL-6 expression in gastrointestinal NETs was higher in patients with lower high-density lipoprotein cholesterol fraction (HDL-C) levels and it positively correlated with the disease progression [159].

## 8. Conclusions

GEP-NETs, due to their heterogeneous morphology, wide and ambiguous clinical manifestation, are often not diagnosed effectively, but with the development of diagnostic methods and the cooperation of medical professionals of various specialties, the statistics of an effective diagnosis improves [160]. Simultaneously, the prevalence of obesity and comorbidities is still increasing worldwide, which is mainly caused by poor eating habits or insufficient physical activity [161]. Although the mechanisms of its influence on the development of neuroendocrine neoplasms are not fully clear, it is possible that the metabolic effects associated with obesity may increase the frequency of GEP-NET prevalence [23,146]. Chronic inflammation, sustained by the presence of pro-inflammatory substances, occurring in obesity with hyperplasia of WAT adipocytes and an increase in the level of free fatty acids, is a phenomenon that may promote cell damage leading to carcinogenesis [10]. It has been suggested that the imbalance of the immune system, particularly disturbed release of pro- and anti-inflammatory factors associated with infiltration of immunomodulatory substances into the tumor environment, may constitute favorable conditions for the development of GEP-NETs [162]. It is important to understand the mechanisms that are likely to influence the development of neoplasm in obesity, which may be a basis for choosing the right direction in the prevention, diagnosis, and therapy of GEP-NETs.

## Figures and Tables

**Table 1:** Research on changes in selected parameters of inflammation in gastroenteropancreatic neuroendocrine tumors (GEP-NETs).

Type of Tumor	Characteristics of Subjects	Parameters	References
GEP-NET	101 patients with GEP-NET and 150 controls (healthy volunteers); analysis of single nucleotide polymorphisms (SNPs)	*IL-2-330 T/G*, statistically significant association between high-expression *GG* genotype and high-expression *G*-allele and risk of GEP-NET	[139]
GEP-NET	101 patients with GEP-NET and 20 controls (healthy volunteers)	↑IL-2 (highest in patients with functional GEP-NET); IL-2 more specific in detecting GEP-NET than CgA and 5-HIAA	[139]
GEP-NEN	43 patients with GEP-NENs	↑TNF-α (correlation with tumor to higher grade and proliferation rates, expression positively correlated with death); ↑IL-6 with tumor grade (not statistically relevant); ↓IL-2, ↓IL-1β	[116]
GEP-NET	80 patients with GEP-NETs and 162 healthy controls; SNP *IL-6-174 C/G* and IL-6 serum values	↑*IL-6-174 CG/GG*-NF-pNET (statistically significant with functional pNET); ↑IL-6 values serum: correlated significantly *GG IL-6-174* genotype in all GEP-NETs, significantly higher IL-6 in NF-pNET with functional-pNET and GI-NET	[117]
pNET	60 patients with pNET and 60 healthy controls; IL-1β SNP (*IL-1β-511-C/T*, *+3954C/T*) and IL-1β serum values	High expression *IL-1β-511-C/T* in functional pNET (statistically significant); −511/+3954 CTCT: risk of developing functional pNET (statistically significant); −511/+3954 CTCC: risk of developing NF-pNET (statistically significant); IL-1β serum levels: undetectable	[121]
GEP-NET	Human NET cell lines: BON (functional pNET, LCC-18 (NF-colorectal NET), QGP (NF-pNET), and cell lines BxPc3 and PANC-1 (human pancreatic carcinoma)	Expression TGF-β1 and its receptor TGF-βRI and TGF-βRII in three tested NET cell lines and in 67% of human NETs (possible inhibition of paracrine and autocrine growth by TGFβ1)	[126]
GEP-NET	38 patients with NENs and 23 healthy controls	↑VEGF vs controls (statistically significant, also a significant increase with tumor progression); ↑angiogenin vs controls (statistically significant); ↑IL-8 vs control (statistically significant); highest level IL-6 in patients which non-survivors compared to survivors	[130]
pNET	52 cases of pancreatic adenocarcinoma and 52 with pNETs	IL-8, IL-8RA; IL-8RB expression in pNET (21%, 63%, 92%): no statistically significant correlation between the expression IL-8, IL-8RA; IL-8RB and tumor grade	[131]
GEP-NET	145 patients with GEP-NETs and 150 healthy controls	↑VEGF in GEP-NETs vs healthy controls (the main with metastatic tumors; statistically significant); ↑VEGF in patients with metastases compared to patients with non-metastatic tumor (statistically significant); *VEGF-1145G* allele more frequent in NF-GI-NET than in controls (not statistically significant); Higher sensitivity VEGF than 5-HIAA but lower than CgA in tumor detection (both pNET and GI-NET); higher VEGF expression in midgut tumors than in foregut tumors (statistically significant difference)	[132]
GEP-NET	58 patients with GEP-NETs; the analysis of SNPs VEGFA, VEGFR2, and VEGFR3	*VEGF-A rs699947C*, *VEGF-A rs2010963GC*, and *VEGFR-3 rs307821C* correlated with an increased risk of disease recurrence;	[133]
NET	115 patients with NETs	A correlation between NLR cut-off value above 3.01 and tumor localization (the main in the lung), higher histological grade, high mitosis, a high Ki-67 proliferation index, distant metastasis, and lymphovascular invasion; no correlations in the case of PLR.	[134]
pNET	172 patients with pNETs undergoing potentially curative resection (166 patients, curative resection and 6 patients, palliative surgery)	↑NLR and ↑PLR significantly associated with shorter overall survival and disease-free survival.	[140]
pNET	165 patients with pNET	A correlation statistically significant between NLR cut-off value >2.4 with tumor size, tumor-node metastasis (stage III and IV), and tumor grade; no correlation NLR with age, gender, location, vessel, and nerve invasion.	[135]
pNETs	95 patients with resectable pNETs	↑NLR is related with advanced tumor stage and higher grade (higher levels NLR in neuroendocrine carcinoma G3 than NET G1 and G2, higher in later stage, tumor thrombus and lymph node metastasis: statistically significant results; NLR independent risk factors for lymph node metastasis (significant differences between groups with and without lymph node metastasis)	[136]
GEP-NET	48 patients with GEP-NETs (foregut, midgut, and hindgut) that underwent resective surgery (curative GI-NET)	NLR >2.6 predicted the presence of peritoneal metastasis (statistically significant); PLR and PWR tended to predict the presence of multifocal disease but statistically insignificant	[137]

Abbreviations used: 5-HIAA: 5-hydroxyindoleacetic acid; CgA: chromogranin A; G1: grade 1 (low-grade tumor), G2: grade 2 (intermediate-grade tumor); G3: grade 3 (high-grade tumor); GI-NET: gastrointestinal tract neuroendocrine tumor; IL-1β: interleukin 1β; IL-2: interleukin 2; IL-6: interleukin 6; IL-8: interleukin 8; IL-8RA/RB: interleukin 8 receptors A/B; NF-GI-NET: nonfunctional gastrointestinal tract neuroendocrine tumor; NF-pNET: nonfunctional pancreatic neuroendocrine tumor; NLR: neutrophil–lymphocyte ratio; PLR: platelet–lymphocyte ratio; pNET: pancreatic neuroendocrine tumor; PWR: platelet-to-white blood cells ratio; SNPs: single nucleotide polymorphisms; TGF-β1: transforming growth factor β1; TGF-βRI, TGF-βRII: TGF-β type I transmembrane receptor/ TGF-β type II transmembrane receptor; TNF-α: tumor necrosis factor α; VEGF or VEGF-A: vascular endothelial growth factor; VEGFR-2/3: vascular endothelial growth factor receptor 2/3. The upwards arrow indicates an increase in the parameter’s level; the downwards arrow indicates a decrease in the parameter’s level.

## Data Availability

Not applicable.
